# Surface soil phytoliths as vegetation and altitude indicators: a study from the southern Himalaya

**DOI:** 10.1038/srep15523

**Published:** 2015-10-26

**Authors:** Xiaohong An, Houyuan Lu, Guoqiang Chu

**Affiliations:** 1 Institute of Geology, Chinese Academy of Geological Sciences, Beijing 100037, China; 2 Key Laboratory of Cenozoic Geology and Environment, Institute of Geology and Geophysics, Chinese Academy of Sciences, Beijing 100029, China; 3 Center for Excellence in Tibetan Plateau Earth Science, Chinese Academy of Sciences, Beijing 100101, China

## Abstract

Phytoliths represent one of the few available altitudinal vegetation proxies for mountain ecosystems. This study analyzed 41 topsoil phytolith samples collected from five altitudinal zones in the southern Himalaya as far as, and beyond, the timberline, from tropical forest (up to 1,000 m a.s.l.) to subtropical forest (1,000–2,000 m a.s.l.), to temperate forest (2,000–3,000 m a.s.l.), to subalpine forest (3,000–4,100 m a.s.l.) and finally to alpine scrub (4,100–5,200 m a.s.l.). The statistical results show a good correlation between phytolith assemblages and these five altitudinal vegetation zones: the five phytolith assemblages identified effectively differentiated these five altitudinal vegetation zones. In particular, coniferous phytoliths accurately indicated the timberline. Additionally, we tested the phytolith index Ic (a proxy for estimating the percentage of Pooideae *vis-à-vis* the total grass content) as a quantifier of phytolith variety *versus* altitude. Ic increased along altitude, as expected. An investigation of phytoliths provided an initial basis for the analysis of the composition of gramineous vegetation. Furthermore, redundancy analysis and discriminant analysis also suggested a significant correlation between phytolith assemblages and altitude. Our research therefore provides an up-to-date analogue for the reconstruction of changes to palaeovegetation and palaeoaltitude in mountainous areas.

Plateau palaeoaltitude has attracted considerable scientific attention, in particular because plateau uplift is an external expression of tectonic movements and a principal factor influencing climate change^1^,^2^, both of which are geoscientifically significant. However, there are few palaeoaltitude indicators for mountain ecosystems. Although pollen can be an effective indicator, and has been used in estimating palaeoaltitude in the Mercantour Massif, the eastern European Alps, the eastern Pyrenees, the Sila Massif, and the northern and central Apennines^2^, difficulties have arisen in distinguishing different species of the same plant family^3^,^4^, and in clarifying the complex transportation and deposition of pollen-spores^3^,^5^, which is vital for palaeoaltitude estimation. In contrast, phytoliths are more resistant than pollen grains to biogenic or physical forces during their deposition, demonstrating that phytolith analysis serve as both an efficient and a complementary route for the study of palaeoaltitude.

Phytoliths are hydrated silicon particles formed in plant growth and preserved in soils and sediments after plant tissues have decayed^6^. They have been proven to be reliable indicators in palaeovegetation and palaeoenvironment reconstruction^7^,^8^,^9^. In mountainous areas like the Himalaya, phytoliths and pollen grains may complement each other, because some deficiencies in pollen-spores can be countered by the presence of phytoliths in the study of the relation between montane vegetation and altitude. First, most phytoliths are naturally resistant to strong weathering and are therefore well-preserved in terrestrial sediments, where pollen-spores can be easily destroyed^10^. Second, even though phytoliths can be transported by the wind, gravity-aided *in situ* deposition remains their dominant *modus operandi*^6^,^10^. Third, phytoliths are more reliable indicators for differentiating varieties of grass subfamily and genera than pollen-spores. Besides, as the study of phytolith morphology progresses, the potential of phytoliths to differentiate various microenvironments, especially near the timberline, has become increasingly apparent^11^,^12^.

There has been some limited research focusing on morphology and phytolith types in mountainous areas^13^,^14^,^15^,^16^, but, as far as we know, only a very few studies of the relation between phytolith composition and altitudinal vegetation belts, *e.g*. on tropical African mountains^17^, have been published. More international research is required. We chose the Himalaya, specifically the southern Qinghai-Tibet Plateau (QTP), for our research.

The QTP averages >4,000 m a.s.l., rising to 8,848 m a.s.l. at Mount Everest (Qomolangma). With its typical mountain climate ecosystem, it is a key area in the scientific research into Asian, and even global, climate change^18^. The uplift of the QTP is also crucial to a better understanding of global tectonics^19^. The study of the QTP’s palaeoaltitude could be significant in both regards, but there remain no direct records of the local palaeoaltitude, palaeovegetation or palaeo-timberline^20^.

The Himalayan Mountains form the southern margin of the QTP and the northern boundary of the Indo-Gangetic Plain (Fig. 1). The southern Himalaya provides environments for various vegetation types, ranging clearly from tropical rainforest to perpetual frost along the altitudinal gradient^21^. It is an optimum area for the study of variations in vegetation, as well research into indicators of QTP palaeoaltitude. Several classification systems are already available for the vegetation of Nepal^22^,^23^,^24^, which lies on the mountain range’s southern flank. Notably, Dobremez *et al.* developed a six-category bio-climatic (altitudinal) classification of vegetation^23^. The published data have focused on flora classification and the characterization of plants^24^,^25^, and the area’s flora and vertical vegetation zones are therefore well-documented. However, due to international borders and poor accessibility, no known work on indexing vegetation belts along the tropical rainforest to perpetual frost altitudinal gradient has been conducted, even if the vertical vegetation range and climate change gradient are most marked in this area. It is therefore imperative to establish a useful index for the reconstruction of both palaeovegetation and palaeoaltitude in this region.

We obtained a diverse collection of samples from a wide range of vegetation belts from the southern Himalaya. In this study, we aimed to explore variations in the composition of phytolith assemblages, and verify our hypothesis that phytolith assemblages can indicate and differentiate vegetation zones along an altitude gradient, providing the basic data necessary for the reconstruction of palaeovegetation and palaeoaltitude in mountainous areas.

## Results

### Vegetation description and material

The Himalaya exhibit typical montane altitudinal vegetation belts. Based on previous work, mainly Dobremez’s altitudinal classification^23^, this paper classifies local forest vegetation vertically from bottom to top into six formations (Fig. 2).

### Tropical moist lowland Indo-Malayan forest (<1,000 m a.s.l.) (Fig. 2a)

*Shorea robusta* (Sal) is predominant in this belt. *Acacia catechu* and *Dalbergia sissoo* replace Sal in riverine forests. Other dominant broadleaved evergreen forest types include *Cycas pectinata*, *Gnetum montanum*, *Calamus* spp., *Cythea spinulosa*, Arecaceae, Magnoliaceae and *Pandanus nepalenses*, except for some coniferous forests (*Pinus roxburgii*) on southern slopes at higher altitudes. In the foothills of western Nepal, Sal forest is replaced by *Terminalia*/*Anogeissus* forest. This zone consists of ~2,000 species of flowering plants and ~80 species of *Pteridophytes*. The common understory grasses include Zingiberaceae, Acanthaceae, Commelinaceae in addition to tall bamboos and grass. Grassland emerges where rainforest has been cut down. Grassland is composed of the tall grasses *Saccharum arundinaccum*, *Apluda mutica*, *Themeda Forssk*, *et al.* Rice, maize and banana are cultivated in this assemblage^21^,^26^.

### Subtropical forest (1,000–2,000 m a.s.l.) (Fig. 2b)

This consists of species such as *Schima wallichii*, *Castanopsis indica* and *Castenopsis tribuloides* in relatively humid areas, and *Pinus roxburghii* forests in drier regions. >1,900 flowering plant species grow vigorously in this zone^27^. *Sinarundinaria nitida* accounts for a large proportion of the total shrub content. The grasses *Carex changmuensis*, *Arisaema* sp., *Ophiopogon bodinieri*, *Elatostema surculosum*, *Miscanthus* sp. *et al.* are also found in these forests^26^. Cultivation is common in this belt. In some places, natural forests have been almost entirely replaced by cultivated crops such as *Musa paradisiaca* and *Chaenomeles sinensis*. Owing to the hot and rainy summer, as well as the dry and warm winter, there are many paddy fields in this zone^28^.

### Temperate forest (2,000–3,000 m a.s.l.) (Fig. 2c)

This zone is dominated by plants such as *Quercus lamillosa* and *Q. semicarpifolia*, forming pure or mixed broadleaved evergreen forest. This latter category can be subdivided into lower temperate mixed broadleaved forest, upper temperate broadleaved forest, upper temperate mixed broadleaved and coniferous forest^22^. Bamboo grows in sunny areas in this zone, and *Sinarundinaria nitida* is abundant^28^. *Ainsliaca latifolia*, *Ophoiopogon intermedia*, *Pilea racemosa*, *Arisaema*, *Zingiber* and *Thalictrum* are common understory grasses^26^.

### Subalpine forest (3,000–4,100 m a.s.l.) (Fig. 2d)

This zone is inhabited by forest vegetation up to the timberline, with >1,400 flowering plants^27^. *Betula*-*Rhododendron campanulatum* and *Abies spectabilis* forests are representative of the vegetation of this zone. *Rhododendron* spp. forms mixed forest within *Abies* or *Betula* forest, or occurs as a component of areas of open shrub. *Juniperus* spp. grows in the drier forest areas of this zone. Herbaceous plants growing in this zone include *Fagopyrum dibotrys*, *Carex lacta*, *Elatostema obtusum*, *Arisaema jasquemontii*, *Poa crymophila*, *Deyeuxia scabrescens*, *Chenopodium* sp., *Oryzopsis lateralis*, *Arundinalla nepalensis*, *Arthraxon* sp., *Pennisctum flaccidum et al.*^26^. The cultivation line reaches as high as 3,600 m a.s.l., where buckwheat is planted^28^. The highest elevation at which *Abies spectabilis*, *Pinus wallichiana* and *B. utilis* grow is 4,000–4,300 m a.s.l.^29^.

### Alpine shrub (4,100–5,200 m a.s.l.)

Alpine shrub is characterized by the presence of various stunted bushy shrubs, including *Juniperus* spp., *Rhododendron* spp., *Juniperus recurva*, *Juniperus indica*, *Juniperus communis*, *Rhododendron anthopogon, Rhododendron lepidotum*, *Ephedra gerardiana* and *Hippophae tibetana*. *Primula* spp., *Gentiana* spp., *Corydalis* spp., *Saussurea* spp., *Kobresia* spp., *Carex* spp., *Ptilagrostis* spp., *Poa* spp., *Deyeuxia* spp., *Festuca* spp., *Danthonia* spp. and *Helictotrichon* spp. are common and important grasses in this zone^27^.

### Perpetual snow (>5,200 m a.s.l.) (Fig. 2e)

This zone is composed of permanent snowfields, rocks, glaciers and ice on the high Himalayan ranges to the north. The area is barren, with lichens on exposed rocky places and a few hardy flowering plants, such as *Stellaria decumbens*. The main vegetation type on the northern slopes of the Himalaya is grass, reflecting the cold-dry climate of the QTP. Forests can be found only in valleys below 4,100 m a.s.l.^28^.

Table S1 shows the coordinates, altitudes and the principal flora of the sampling sites.

### Phytolith types

Of the 47 processed samples, 41 contained >300 phytoliths. These were identified and classified into the 27 common categories listed below, mainly following the classification system used by Lu *et al.*^30^, but with reference to the classification systems of Wang and Lu^31^, Kondo *et al.*^32^ and Twiss *et al.*^33^, and using the International Code for Phytolith Nomenclature 1.0^34^.

The woody phytolith types include globular, abbreviated stellate, cylindrical sclereid and Gymnosperm. Globular types were subdivided into globular echinate (Fig. 3a) and globular granulate (Fig. 3b), the former being produced specifically in Palmae and the latter in tropical trees and shrubs (but principally in Palmae in China)^14^,^35^,^36^. Abbreviated stellate (jigsaw) phytoliths (Fig. 3x) are produced by evergreen broadleaved plants^13^,^31^,^37^,^38^. The cylindrical sclereid (Y-shape) phytolith (Fig. 3aa) is a type particular to broadleaved plants^31^,^38^. Gymnosperm (Fig. 3w), as the term suggests, derives mainly from Pinaceae^39^,^40^.

A diversity of gramineous phytolith types were observed during the course of this study. Bilobate short cell (dumbbell^30^) (Fig. 3d) and cylindrical polylobate (multilobate) phytoliths (Fig. 3f) are both representative of Panicoideae, which adapt to warm-humid conditions^31^. The cross-shaped^41^ phytolith (Fig. 3c) is typical of the bilobate short cell type; the form of this phytolith produced in maize can be differentiated from the type produced in wild grass by its mirror-image and greater width (usually >12.5μm)^42^,^43^. The cross-shaped type referred to in this paper specifically represents maize, with other variants classed as bilobate short cell phytoliths. Square saddle (short saddle^30^) phytoliths (Fig. 3h) are mainly found in Chloridoideae, with a small fraction occurring in Arundinoideae. Chloridoideae mainly grows in dry-hot conditions. Arundinoideae covers a broad range of southern China. The square saddle type is considered representative of C_4_ plants^31^,^38^. Oblong concave saddle (long saddle^30^) phytoliths (Fig. 3I,j) are produced in Bambusoideae, which grow in hot, moist climates throughout southern China^31^,^44^.

Cuneiform bulliform cell (fan-shaped^30^) (Fig. 3o–3q) and parallepipedal bulliform cell (square and rectangular^30^) (Fig. 3u,v) phytoliths develop from motor cells produced in Panicoideae, Oryzoideae and Bambusoideae^31^. These plants flourish in the warm and humid climate of southeastern China. Sometimes an apparently parallepipedal bulliform cell can actually be the side-on view of a cuneiform bulliform cell^31^. Some Chloridoideae produce parallepipedal bulliform cells, but not cuneiform bulliform cells. Cuneiform bulliform cell-rice (Fig. 3o) and cuneiform bulliform cell-bamboo (Fig. 3p) phytoliths can be differentiated from other cuneiform bulliform cell phytoliths by the ornamentation of cracks and spines along their front edges, respectively^31^,^45^.

The hair cell (point^30^) phytolith type (Fig. 3t) develops from spiny grass cells, which resist cold and drought. Hair cell phytoliths thrive in northern and northwestern China^31^; rondel (Fig. 3g), trapeziform (Fig. 3r,s) and *Stipa*-bilobate short cell (Fig. 3e) phytoliths are produced in Pooideae^38^, and are representative of cold climates and high altitudes within tropical regions. *Stipa*-bilobate short cell phytoliths can be differentiated from Panicoideae bilobate short cell phytoliths by their slim necks and differing opposite sides^15^,^46^,^47^.

In addition to the abovementioned, the Pteridophyte phytolith type (Fig. 3ab) is particular to ferns^31^, and the sedge (papillae^17^) type (Fig. 3m,n) is typical of Cyperaceae, a grass-like plant growing in wet places^31^.

We identified some types with no current taxonomical significance: the one-horned tower (Fig. 3k) and two-horned tower (Fig. 3l) are small phytolith types, exhibiting a diversity of shapes^48^; the elongate type, including elongate smooth (Fig. 3z) and elongate echinate (Fig. 3y) phytoliths, develop from long epidermis cells^31^ (these increase in quantity in China from south to north and from humid to dry regions); and the gobbet (nubby-irregular shape) type, a non-gramineous phytolith, appears in arid areas in China^30^. We have classified these difficult-to-categorize types as unknown and as-yet-uninvestigated (see Supplementary Fig. S5 online).

Supplementary Table S2 (online) is a summary of phytolith types, descriptions and their source plants and ecoenvironments. More plates of the aforementioned phytolith types can be found in Supplementary Figs S1-S5 (online).

### Phytolith assemblages

Phytoliths were divided into the following five assemblages, according to phytolith type and percentage of total composition (Fig. 4).

Assemblage I, from tropical lowland evergreen broadleaved forest (<1,000 m a.s.l.), is characterized by a high percentage of cylindrical sclereid, globular and cuneiform bulliform cell phytoliths. The maximum contents of abbreviated stellate, cylindrical sclereid, globular and cuneiform bulliform cell phytoliths are 1.8%, 7.3%, 3.9% and 19.6% of the total phytolith content, respectively. The highest numbers of broadleaved types (cylindrical sclereid and abbreviated stellate) are found in this belt, where evergreen broadleaved plants predominate. The volumetric production of Palmaceae phytoliths implies tropical lowland, high temperature conditions. Parallepipedal bulliform cell 1, bilobate short cell, and oblong concave saddle types account for 11.6%, 5.2%, 3.4% and 4.3% of the content total, respectively. The low contents of gramineous types such as bilobate short cell and oblong concave saddle phytoliths indicate a weak growth of grass and bamboo in low altitude areas with abundant tree cover. Sedge (2.5%) is at its maximum in this zone, implying a lowland, humid environment. There is also a high quantity of unknown types (4.8%); many varieties from this wide range of phytolith morphotypes have yet to be investigated. The emergence of cuneiform bulliform cell-rice (0.5%) and maize cross (0.5%) phytoliths implies that rice and maize were cultivated in this low altitude zone.

Assemblage II, from subtropical broadleaved forest (1,000–2,000 m a.s.l.), is characterized by cylindrical sclereid, bilobate short cell and cuneiform bulliform cell phytoliths. The higher percentages of bilobate short cells (15.8%) and oblong concave saddles (11.8%) *vis-à-vis* total content in this assemblage compared to Assemblage I suggests a greater quantity of grasses and bamboo. Frequent and repeated human cultivation has resulted in a high grass content^28^. In contrast with gramineous types, Palmaceae (0.9%) and cylindrical sclereid (2.6%) phytoliths decrease markedly, and abbreviated stellate types decline sharply from 1.8% to 0.3% of total content, but cylindrical sclereid remains the principal woody type. Both falls in overall content are likely to be the result of a decrease in woody plants due to human cultivation.

Assemblage III, from warm-temperate mixed forest (2,000–3,000 m a.s.l.), is characterized by saddle, parallepipedal bulliform cell and rondel phytoliths. The increase in rondel (2.5% to 5.8%) and trapeziform (0.8% to 2.4%) phytoliths, and the decrease in bilobate short cells (15.8% to 4%), probably reflects the change in climate from warm to cold, and in altitude from low to high. The high altitude type of *Stipa*-bilobate short cell phytolith appears. Cuneiform bulliform cell-rice and cross-shape phytoliths were not observed.

Assemblage IV, from subalpine cold-temperate needle-leaved forest (3,000–4,100 m a.s.l.), is characterized by gymnosperm-type, trapeziform and *Stipa*-bilobate short cell phytoliths. Gymnosperm-type phytolith content increases from 0.2% to 8.6%, *Stipa*-bilobate short cell phytolith content from 2.3% to 5.6%, and trapeziform phytolith content from 2.4% to 16.2% *vis-à-vis* Assemblage III, while oblong concave saddle phytoliths decrease from 16.9% to 1.3%, and parallepipedal bulliform cell 1 types decline from 15.1% to 4.8%, indicating flourishing gymnosperm forest and understory Pooideae environments, both of which are indicative of high altitude conditions.

Assemblage V, from alpine shrub (4,100–5,200 m a.s.l.), is characterized by trapeziform, rondel and gobbet phytoliths. The percentages of most types drop very low in this assemblage, but there are abundant rondel (5.6%), trapeziform (30.6%) and gobbet (6.9%) phytoliths, indicating the predominance of high altitude, cold climate-adaptable plants.

To summarize, an abundance of phytolith types, accompanied by clear variations in phytolith percentages *vis-à-vis* total phytolith content, occurs along the altitudinal gradient on the slopes of the southern Himalaya. Phytolith assemblages can be clearly differentiated (Table 1).

### Floristic composition (RDA results)

18 of the aforementioned 27 phytolith types account for a certain proportion of the samples and clearly indicate vegetation type. In elucidating the relation between phytolith assemblages and altitude, redundancy analysis (RDA) results (Fig. 5) show that the first and second axes account for 42% and 16% of the total variance, respectively, describing 58% of the information in total. The other axes exhibit very low values. This suggests that phytolith composition is controlled by environmental factors represented by the first two axes, and especially by altitude (the first axis).

As the RDA results reveal, low altitude-adaptable phytolith types are grouped in a positive direction toward the first axis, while those adapting to high altitude point in a negative direction. The former group includes globular, cylindrical sclereid, bilobate short cell, parallepipedal bulliform cell, cuneiform bulliform cell, cuneiform bulliform cell-bamboo, oblong concave saddle and square saddle phytoliths; the latter is composed of gymnosperm-type, hair cell, elongate smooth, elongate echinate, rondel, trapeziform, *Stipa*-bilobate short cell and gobbet phytoliths (Fig. 5a). Low altitude samples t1–t20 are positively grouped toward the first axis, while high altitude samples t21–t41 exhibit a negative direction (Fig. 5b). This reflects the clustering of phytolith samples from higher altitudes around negative coordinates, in contrast to samples from lower altitudes, which are clustered around positive coordinates. This therefore demonstrates a correspondence between phytolith assemblages and the altitudinal distribution of vegetation.

### Floristic composition (DA results)

Discriminant analysis (DA) was applied to test whether the established surface phytolith assemblages described in this paper can reliably differentiate the vertical vegetation belts in the southern Himalaya. Using *a priori* groups, the 41 surface samples with the 18 phytolith types used for RDA were then classified to co-validate the classification of phytolith assemblages. 39 samples (95%) were correctly classified with respect to these *a priori* groups (Table 2). The first two discriminant function scores are illustrated herein: group centroids are distinctly separate (Fig. 6). The classification function coefficients are shown in supplementary Table S3 (online).

### Phytolith indices

In order to present a direct and quantifiable relation between altitude and phytolith type, we applied the phytolith indices Ic and Iph^17^. Ic represents the proportion of short cell phytoliths from Pooideae relative to total short cell phytoliths from Pooideae, Chloridoideae and Panicoideae^17^, thus:


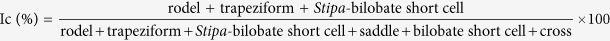


Ic (%) represents an index for the total percentage of Pooideae grass cover.

Ic has been shown to depend principally on altitude (SPSS curve estimation of R^2^ = 0.8) (Fig. 7a). As a result, it is clear that, along with increasing altitude, Pooideae grasses increase gradually, while Chloridoideae and Panicoideae grasses decrease. The relation between altitude and Ic can be expressed using the equation:


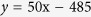


where x represents Ic (%) and y represents elevation (m).

Ic is a climatic index for temperature, because a high Ic represents higher quantities of Pooideae, which adapt to the cool climates prevalent at high altitudes^49^. Altitude is the most influential factor *vis-à-vis* temperature in the Nepal Himalaya; mean annual temperature in general gradually decreases northward as altitude increases^50^. The Ic results therefore render an approximate, linear correlation between Pooideae amount, temperature and altitude^51^.

Iph was also tested, but the result of the curve estimation was less useful, because the sig. = 0.109 for curve estimation (Fig. 7b).

## Discussion

We have obtained samples from the world’s highest mountain range, the Himalaya, and have derived significant results from our analysis of the material. Although the composition of preserved phytoliths in soils is influenced by translocation and dissolution^52^, phytolith assemblages in soils reflect the composition of local vegetation^10^. First, the surface soil phytoliths described in this paper are mainly bleached mountain spodosols, dark-brown mountain earths and brown mountain earths^21^. These weakly acidic soils are suitable depositional environments for phytoliths; pH ranges between four and six, and is therefore never high enough for the sustained dissolution of phytoliths. Even if dissolved, dissolution rates are similar for most phytolith morphotypes^53^,^54^. Second, small phytoliths may be dispersed by the wind^55^. However, the geographical scale of transfer *versus* study area is considered insignificant in this paper. Most probably, therefore, phytolith composition accurately reflects phytolith composition for each corresponding vegetation belt.

It should however be recognized that the percentage content of each phytolith *vis-à-vis* total phytolith content cannot be equal to the percentage content of the corresponding vegetation. Some plants yield abundant phytoliths, resulting in over-representation. So in describing vegetation assemblages, it is imperative we focus on the relative variation in each phytolith type *versus* altitude. Furthermore, the taphonomy of phytoliths in sediments should be considered when reconstructing palaeovegetation^10^.

In view of the reliability of our samples, the phytoliths analyzed in this paper can accurately describe vegetation zones. Phytolith percentages clearly illustrate that each vegetation zone has a distinctive phytolith assemblage and some typical phytolith types. In tropical forest at low altitudes (<1,000 m a.s.l.), the cylindrical sclereid/globular/cuneiform bulliform cell phytolith assemblage corresponds to an abundance of hot climate-adaptable woody plants, such as *Shorea*, *Cycas*, Magnoliaceae, Palmae, and Panicoideae, principally *Saccharum*, *Apluda* and *Themeda.* In the subtropical broadleaved forest belt (1,000–2,000 m a.s.l.), the cylindrical sclereid/bilobate short cell/cuneiform bulliform cell assemblage highlights the dominance of broadleaved woody plants and tall Panicoideae grasses (*e.g*. *Miscanthus*). In warm-temperate mixed forest (2,000–3,000 m a.s.l.), the saddle/parallepipedal bulliform cell/rondel phytolith assemblage corresponds to the mixture of warm and cold temperate zone grasses, as well as a mixture of broadleaved and coniferous plants. In the subalpine needle-leaved forest zone (3,000–4,100 m a.s.l.), the gymnosperm-type/trapeziform/*Stipa*-bilobate short cell phytolith assemblage suggests the dominance of Gymnosperm (*Abies* and *Pinus*) and low temperate gramineous types (*Deyeuxia* and *Oryzopsis*). In the alpine shrub zone (4,100–5,200 m a.s.l.), an abundance of rondel, trapeziform and gobbet phytoliths implies an absence of woody plants and a predominance of high altitude-adaptable Pooideae, including *Carex*, *Festuca*, *Helictotrichon*, and especially *Ptilagrostis* (belonging to Stipeae).

To corroborate the increasing/decreasing trend in different phytoliths along altitude, typical bilobate short cell, cuneiform bulliform cell and trapeziform phytolith types are shown in boxplots, with emendations (Fig. 8). It is clear that the bilobate short cell and cuneiform bulliform cell types decrease with altitude, while the trapeziform type increases.

DA results, with their clearly-separated group centroids, also demonstrate that the five phytolith assemblages can accurately delineate vertical vegetation zones, as each vegetation belt is represented by a distinctive phytolith assemblage. Our classification of phytolith assemblages suggests that phytoliths can be used as valid proxies for subdividing montane vegetation in the QTP area, and can thus be used effectively in reconstructions of palaeovegetation and palaeoaltitude.

Notably, some phytoliths exhibit a high degree of sensitivity to environment and climate. In particular, the fluctuation of the timberline is crucial to montane phytolith study. It not only reflects variations in vegetational composition, but, more importantly, indicates changes in altitude. The upper altitudinal limit of montane needle-leaved plants is commonly considered to be the same as the timberline^56^. However, most conifer pollens have two or more sacs, *e.g.* pine pollen. Long-distance dispersal of pollens leads to their wide representation in assemblages. Investigations of modern pollens have shown that pine forest only grows where conifer pollens account for at least 30% of the total^4^, so the existence of conifer pollens does not necessarily accurately reflect the proportion of coniferous plants. Autochthonous or proximal deposition is characteristic of phytoliths^31^,^37^. Coniferous phytoliths from the southern slopes of the Himalaya appear only in the planting zone of needle-leaved plants, correspondent to the distribution of coniferous forests; phytoliths can thus be used as discriminants of the timberline and the altitude of montane vegetation zones. When researching montane palaeoenvironments, both the frequency of occurrence and the variability in the content of coniferous phytoliths can provide a good basis for deducing the historical position of the timberline, as well as palaeoaltitude.

Furthermore, crop phytoliths can reflect different types of farming activity^43^,^45^, and indeed different crops grow at different altitudes. The cross-shaped and cuneiform bulliform cell-rice phytoliths produced in the surface soils of the Nepal Himalaya indicate the planting of maize and rice, in accordance with the area’s relatively low altitude. This also implies that human disturbance has changed the composition of the local vegetation. The existence of cultivated phytolith morphotypes in sediments and/or archaeological remains would certainly indicate an historical development of this region by humans^10^.

Based upon our qualitative analysis, we conducted a quantitative analysis, with good results. The mathematical transfer function can be ideal for providing a basis for palaeoenvironmental research, especially when substantial quantities of modern data are applied. In this study, although we did not have a huge number of samples, our Ic values expressed the functional relation between phytolith assemblage variety and altitude very well, demonstrating that Ic is a perfect index for representing gramineous composition^17^ in mountainous areas like the Himalaya. However, we adjusted its formulaic expression. In previous research, the *Stipa*-bilobate short cell phytolith was added to neither the numerator nor the denominator in the index’s mathematical expression. However, *Stipa* belongs to Pooideae, and can survive in extremely cold and arid environments. *Stipa* grasses are widely distributed at high altitudes on the QTP, and are a vital component of the grass cover in our study area^57^. When *Stipa*-bilobate short cells are taken into account, the curve estimation of R^2^ = 0.8. However, R^2^ = 0.7 without the inclusion of *Stipa*-bilobate short cells. Certainly, *Stipa*-bilobate short cells, as an individual phytolith type, should be distinguished from the sum of phytoliths during identification and this should be taken into account when using the Ic index. However, when samples are collected from soil surfaces or Cenozoic stratigraphic sections in cold and arid areas like the QTP, the presence of *Stipa*-bilobate short cells must be fully taken into consideration.

Ic can be used to reconstruct the Quaternary palaeoelevation of the QTP during periods of relatively stable climate. We noted that in the East African tropical mountains, Ic values are ~40, ~70 and ~98 at 2000 m a.s.l., 3000 m a.s.l. and 4000 m a.s.l., respectively^17^. The corresponding values are ~50, ~70 and ~90 in our paper. This may imply that Ic has some universal applicability in low latitude areas, as both of the abovementioned areas are low latitude regions.

The humidity-aridity index Iph was also tested, but proved not obviously applicable to altitude. First, unlike temperature, precipitation in the Nepal Himalaya is affected by two major atmospheric circulation systems. The interaction of the complex topography with the monsoonal and westerly weather systems results in variations in the spatial distribution of rainfall^50^, suggesting that the annual precipitation pattern is not dominated by altitude. Second, Iph was defined as the ratio of Chloridoideae (saddle) *versus* Chloridoideae and Panicoideae (saddle, cross-shaped and bilobate short cell)^17^. The square saddle phytolith is typical type of Chloridoideae. Some Chloridoideae species, which have adapted to drought conditions, are prone to produce square saddle phytoliths. Other Chloridoideae species in humid environments can produce another short cell phytolith, *i.e.* bilobate. Iph has been shown to be applicable to the Great Plains of North America as well as to tropical savannah^17^,^58^; both these environments have dry seasons which last several months. However, the northern Himalaya enjoys rainier weather. Square saddle phytoliths may therefore not indicate the presence of Chloridoideae in the area. Moreover, the presence of Bambusoideae may also render Iph inapplicable. Bambusoideae produce great quantities of oblong concave saddle phytoliths, which account for a certain proportion of all saddle phytoliths. Using Iph as a index, saddle phytoliths should present as Chloridoideae, but the saddle phytoliths in the northern Himalaya are derived from Chloridoideae, Bambusoideae and Arundinoideae. Bremond *et al.* (2008) also demonstrated that Iph was not a relevant proxy for Chloridoideae *versus* Chloridoideae and Panicoideae^17^.

Less research has been conducted into woody phytoliths than gramineous phytoliths, most probably because of the great variety in the morphology of woody phytoliths and the consequent difficulties discriminating between them. If a more detailed identification were possible, the environmental marker function would become more precise. For example, palmaceae phytoliths from the montane subtropical evergreen belt provide good indices for discriminating palm tree planting. There is a fundamental need for systematic and detailed research into woody phytoliths. Such palaeoenvironmental research would provide a basic reference tool for identifying vegetation belts, phytocoenoses, edificators, and even typical species.

## Methods

**Figure 1:**
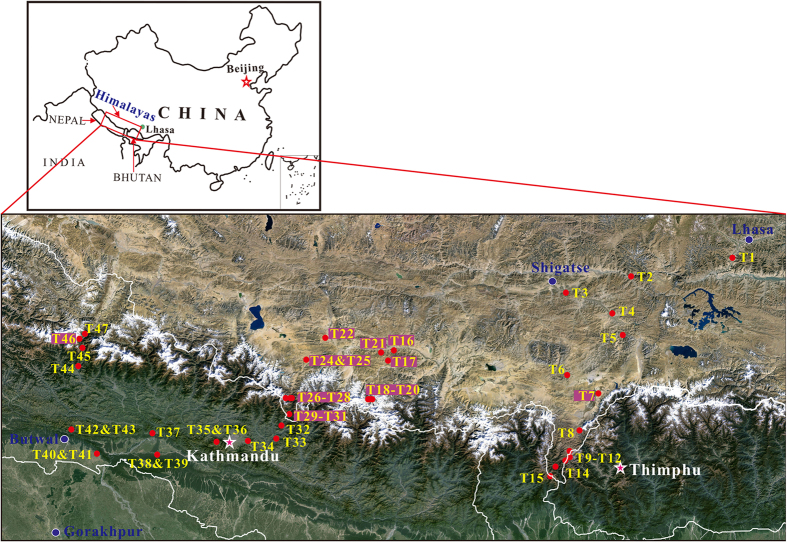
Map of the location of the studied area in the Himalaya between China and Nepal. Red-filled circles show sampling sites. Topographic relief was obtained using a dataset provided by the Geospatial Data Cloud, Computer Network Information Center, Chinese Academy of Sciences ( http://www.gscloud.cn). The figure was created by Xiaohong An using DIVA-GIS 7.5.0 ( http://www.diva-gis.org/).

**Figure 2:**
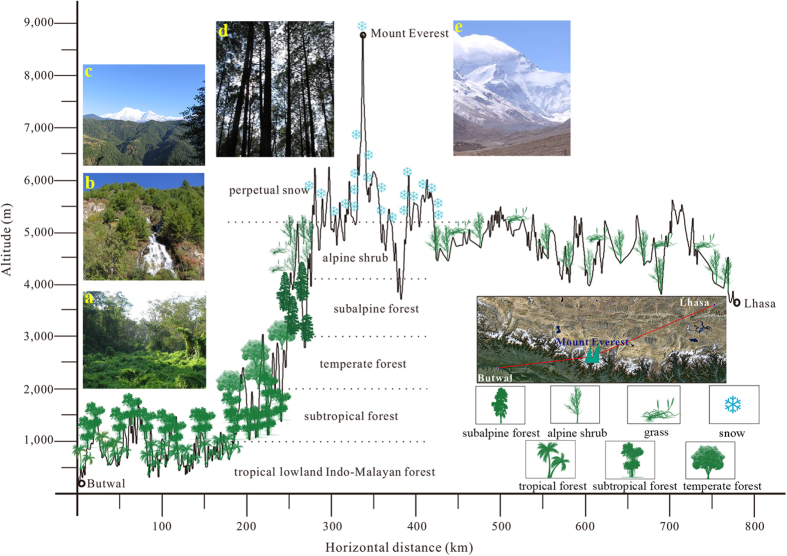
Sketch map of vegetation distribution from Butwal in Nepal to Lhasa on the QTP. Photographs labelled (**a–d**) and (**e**) show tropical forest, subtropical forest, temperate forest, subalpine forest and perpetual snowy mountains, respectively. Topographic relief was provided by the Geospatial Data Cloud, Computer Network Information Center, Chinese Academy of Sciences ( http://www.gscloud.cn). The figure was drawn by Xiaohong An using Photoshop CS6. The photographs were taken by Guoqiang Chu.

**Figure 3:**
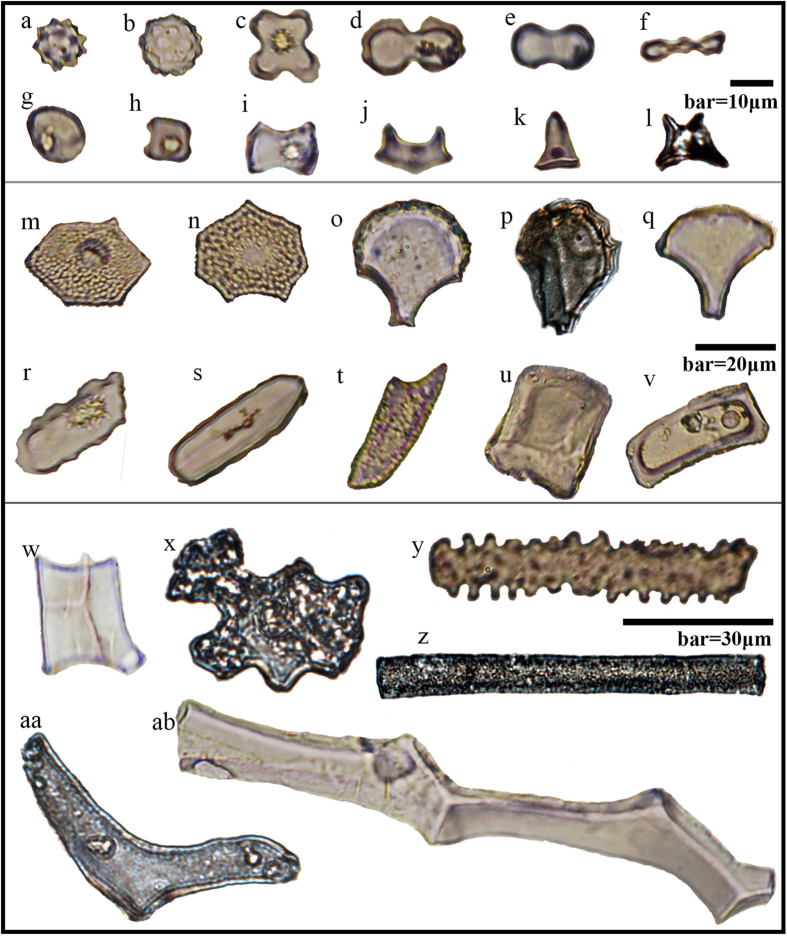
Common phytolith types in surface soils from the southern Himalaya. (**a**) globular echinate; (**b**) globular granulate; (**c**) cross-shaped; (**d**) bilobate short cell; (**e**) *Stipa*-bilobate short cell; (**f**) palylobate; (**g**) rondel; (**h**) square saddle; (**i**) oblong concave saddle 2; (**j**) oblong concave saddle 1; (**k**) one-horned tower; (**l**) two-horned tower; (**m,n**) sedge-type; (**o**) cuneiform bulliform cell-rice; (**p**) cuneiform bulliform cell-bamboo; (**q**) cuneiform bulliform cell; (**r,s**) trapeziform; (**t**) hair cell; (**u**) parallepipedal bulliform cell 1; (**v**) parallepipedal bulliform cell 2; (**w**) gymnosperm-type; (**x**) abbreviated stellate; (**y**) elongate echinate; (**z**) elongate smooth; (**aa**) cylindrical sclereid; (**ab**) pteridophyte.

**Figure 4:**
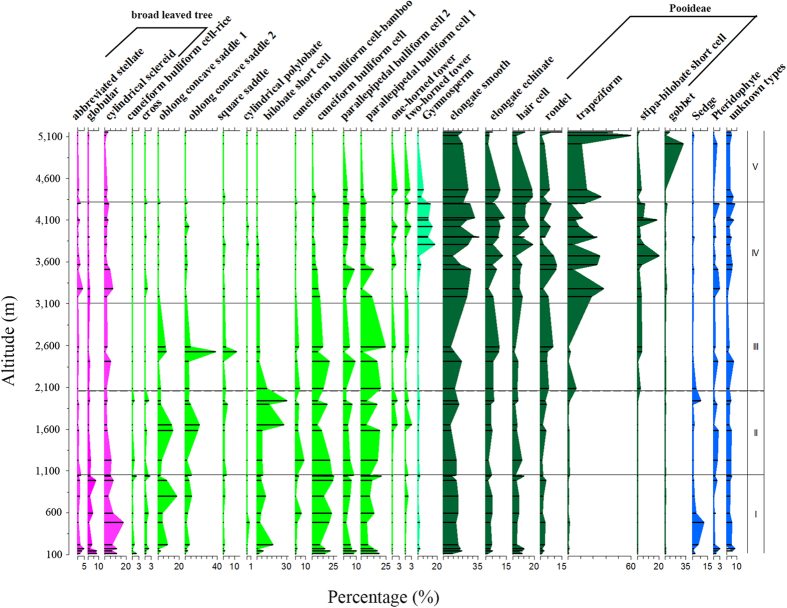
Total content percentages for the five topsoil phytolith assemblages from the southern flanks of the Himalaya. (**I**) the cylindrical sclereid/globular/cuneiform bulliform cell assemblage; (**II**) the cylindrical sclereid/bilobate short cell/cuneiform bulliform cell assemblage; (**III**) the saddle/parallepipedal bulliform cell/rondel assemblage; (**IV**) the gymnosperm-type/trapeziform/*Stipa*-bilobate short cell assemblage; and (**V**) the trapeziform/rondel/gobbet assemblage.

**Figure 5:**
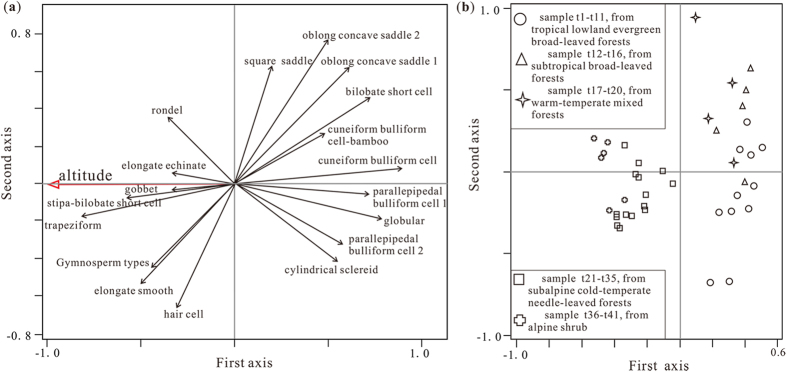
RDA results for principal phytolith types (a) and sampling sites (b).

**Figure 6:**
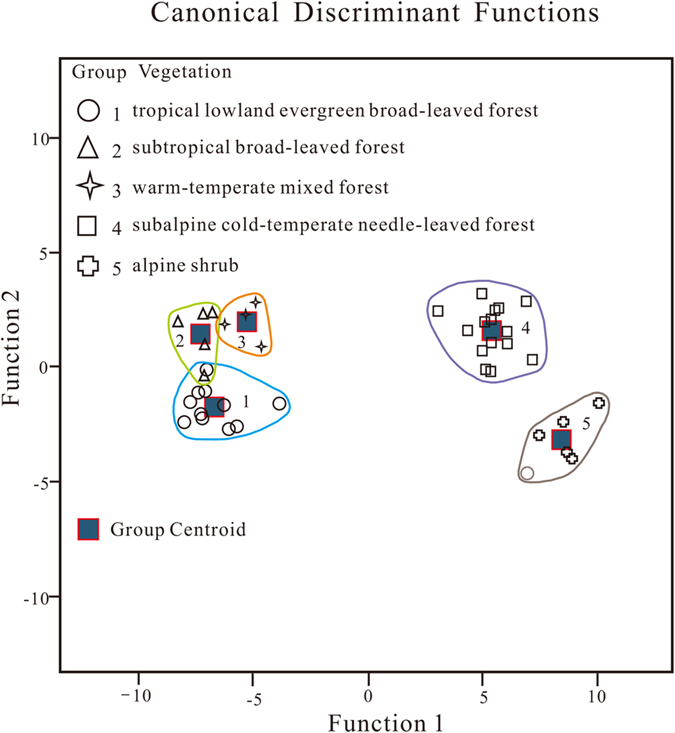
Ordination of the 41 surface samples plotted against canonical discriminant functions 1 and 2. Samples were categorized into five groups according to the five vegetation belts (indicated by different geometric figures).

**Figure 7:**
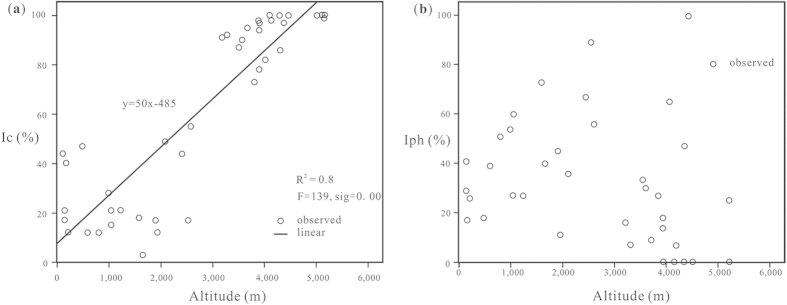
Curve estimation results for Ic (a) and scatter plots of Iph (b). The determination coefficient for Ic is R^2^ = 0.8.

**Figure 8:**
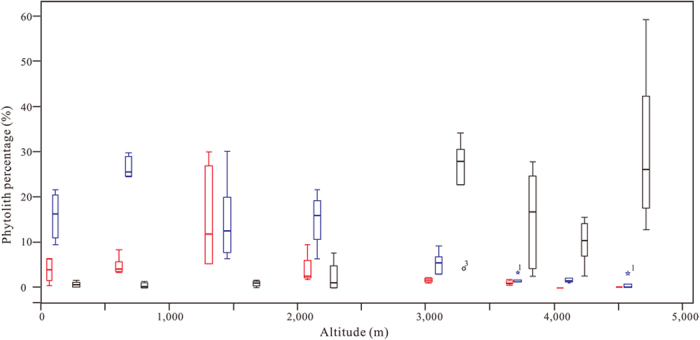
Boxplots of phytolith percentages. Red bars refer to bilobate short cells; blue bars to cuneiform bulliform cells and black bars to trapeziform.

In this study, 47 topsoil samples were collected at altitudes between 100 m a.s.l. and 5200 m a.s.l. from the Sino-Nepal Himalaya (Fig. 1). No sample was collected from the perpetual snow zone, because the vegetation becomes extremely sparse above 5200 m a.s.l. After clearing away any loose debris, each sample was extracted from the top 0–2 cm of surface soil and put into a valve bag. A GPS receiver was applied to keep a record of the longitude, latitude and altitude of each sampling site. Samples were dried out in the laboratory before long-term storage in order to prevent clumping.

Phytoliths were extracted from each sample by conventional heavy liquid flotation based on the method of Piperno and Pearsall^6^,^59^, but were not passed through a sieve, as some phytoliths in this paper (*e.g.* rondel and square saddle phytoliths) are very small and could be filtered off during the process. All samples were treated in the Key Laboratory of Cenozoic Geology and Environment, Institute of Geology and Geophysics, Chinese Academy of Sciences. A subsample of ~1–3 g was taken from each sample, according to its particular composition. Each subsample was sequentially processed as follows: (1) organics were removed with 30% H_2_O_2_; (2) a tablet of *Lycopodium* spores (27637 spores per tablet) was added to determine phytolith concentration (the method of Piperno and Pearsall does not include this step); (3) carbonates were dissolved with 10% HCl; (4) flotation of phytoliths was accomplished using a ZnBr_2_ solution (density 2.35 g/cm^3^); and (5) after cleaning, each subsample was dipped in resinene to facilitate slicing. Morphotypes were counted under a microscope; 41 samples were found to include >300 phytoliths.

Canoco5 software was used to perform RDA^60^.

We used SPSS 17.0 statistics software to build scatter diagrams of Ic and Iph along altitude, and make curve estimations. We also applied SPSS 17.0 to DA and to our boxplots.

## Additional Information

**How to cite this article**: An, X. *et al.* Surface soil phytoliths as vegetation and altitude indicators: a study from the southern Himalaya. *Sci. Rep.* **5**, 15523; doi: 10.1038/srep15523 (2015).

## Supplementary Material

Supplementary Information

## Figures and Tables

**Table 1:** Correlation between phytolith assemblage and vegetation zone.

Altitude	Vegetation Zone	Understory Grasses	Phytolith Assemblage
<1,000 m a.s.l.	Tropical lowland evergreen broadleaved forest	Zingiberaceae, Acanthaceae, Commelinaceae, *Saccharum arundinaccum*, *Apluda mutica*, *Themeda Forssk*, *et al.*	Cylindrical sclereid/globular/ cuneiform bulliform cell assemblage
1,000–2,000 m a.s.l.	Subtropical broadleaved forest	*Carex changmuensis*, *Arisaema* sp., *Ophiopogon bodinieri*, *Elatostema surculosum*, *Miscanthus* sp. *et al.*	Cylindrical sclereid/bilobate short cell/cuneiform bulliform cell assemblage
2,000–3,000 m a.s.l.	Warm-temperate mixed forest	*Ainsliaca latifolia*, *Ophoiopogon intermedia*, *Pilea racemosa*, *Arisaema*, *Zingiber* and *Thalictrum et al.*	Saddle/parallepipedal bulliform cell/rondel assemblage
3,000–4,100 m a.s.l.	Subalpine cold-temperate needle-leaved forest	*Fagopyrum dibotrys*, *Carex lacta*, *Elatostema obtusum*, *Arisaema jasquemontii*, *Poa crymophila*, *Deyeuxia scabrescens*, *Chenopodium* sp., *Oryzopsis lateralis*, *Arundinella nepalensis*, *Arthraxon* sp., *Pennisctum flaccidum et al.*	Gymnosperm-type/ trapeziform/*Stipa*-bilobate short cell assemblage
4,100–5,200 m a.s.l.	Alpine shrub	*Primula* spp., *Gentiana* spp., *Corydalis* spp. , *Saussurea* spp., *Kobresia* spp., *Carex* spp., *Ptilagrostis* spp., *Poa* spp., *Deyeuxia* spp., *Festuca* spp., *Danthonia* spp. and *Helictotrichon* spp. *et al.*	Trapeziform/rondel/gobbet assemblage

**Table 2:** DA results for the 41 surface samples extracted from the five altitudinal vegetation zones.

Actual Group	GroupNo.	Predicted Group Membership					
		1	2	3	4	5	Total
Tropical lowland evergreen Broadleaved forest	1	10 (90.9%)	1 (9.1%)	0	0	0	11
Subtropical broadleaved forest	2	1 (20.0%)	4 (80.0%)	0	0	0	5
Warm-temperate mixed forest	3	0	0	4 (100.0%)	0	0	4
Subalpine cold-temperate needle-leaved forest	4	0	0	0	15 (100.0%)	0	15
Alpine shrub	5	0	0	0	0	6 (100.0%)	6

(95.1% of originally-grouped cases correctly classified).
