# Supplementary material for: Surface soil phytoliths as vegetation and altitude indicators: a study from the southern Himalaya
Source: Sci Rep. 2015 Oct 26;5:15523. doi: 10.1038/srep15523 (PMC4620457; doi:10.1038/srep15523)
Supplement: Supplementary Information [file srep15523-s1.doc]

Supplementary Information for:

# Surface soil phytoliths as vegetation and altitude indicators: a study from the southern Himalaya

Xiaohong An^1, 2^*, Houyuan Lu^2, 3^*, Guoqiang Chu^2^

^1^ Institute of Geology, Chinese Academy of Geological Sciences, Beijing, 100037, China

^2^ Key Laboratory of Cenozoic Geology and Environment, Institute of Geology and Geophysics, Chinese Academy of Sciences, Beijing, 100029, China

^3^ Center for Excellence in Tibetan Plateau Earth Science, Chinese Academy of Sciences, Beijing 100101, China

* Authors to whom correspondence should be addressed

[houyuanlu@mail.iggcas.ac.cn](mailto:houyuanlu@mail.iggcas.ac.cn) (H.Y.L.)

[kja0@163.com](mailto:kja0@163.com) (X.H.A.)

**This file includes:**

**Table S1**

**Figure S1**

**Figure S2**

**Figure S3**

**Figure S4**

**Figure S5**

**Table S1** Coordinate and altitude of the sampling sites, and flora pattern of some sites.

| **Sample Number** | **Site Number** | **Latitude** | **Longitude** | **Altitude** | **Vegetation Type** |
| --- | --- | --- | --- | --- | --- |
|  | T18 | 28°8'26" | 86°51'6" | 5161 |  |
| t41 | T19 | 28°8'22" | 86°51'3" | 5152 | meadow |
| t40 | T20 | 28°8'22" | 86°51'3" | 5152 | meadow |
| t39 | T24 | 28°31'7" | 86°10'0" | 5110 |  |
| t38 | T17 | 28°30'59" | 87°3'48" | 5015 |  |
|  | T23 | 28°43'29" | 86°22'21" | 4595 |  |
| t37 | T8 | 27°49'33" | 89°9'24" | 4465 | shrub |
|  | T7 | 28°10'54" | 89°22'13" | 4445 |  |
| t36 | T6 | 28°21'33" | 89°1'44" | 4377 |  |
| t35 | T15 | 27°23'13" | 88°49'53" | 4303 |  |
| t34 | T22 | 28°43'29" | 86°22'21" | 4294 |  |
|  | T21 | 28°35'20" | 86°59'22" | 4279 |  |
|  | T16 | 28°36'39" | 87°7'49" | 4236 |  |
| t33 | T9 | 27°37'19" | 89°2'21" | 4131 | coniferous forest |
| t32 | T5 | 28°44'18" | 89°39'10" | 4104 |  |
| t31 | T4 | 28°57'18" | 89°32'45" | 4020 |  |
| t30 | T25 | 28°31'7" | 86°10'1" | 3904 |  |
| t29 | T3 | 29°9'33" | 89°1'55" | 3899 |  |
| t28 | T11 | 27°35'19" | 89°2'9" | 3898 | coniferous forest |
| t27 | T10 | 27°35'19" | 89°2'9" | 3893 | coniferous forest |
| t26 | T2 | 29°18'9" | 89°45'38" | 3809 |  |
| t25 | T26 | 28°8'43" | 85°58'43" | 3678 |  |
| t24 | T12 | 27°33'12" | 89°0'15" | 3570 | coniferous forest |
| t23 | T1 | 29°28'5" | 90°55'37" | 3505 |  |
| t22 | T27 | 28°8'43" | 85°5'43" | 3278 | mixed coniferous broad leaved forest |
| t21 | T14 | 27°28'39" | 88°53'23" | 3188 |  |
| t20 | T28 | 28°8'43" | 85°58'44" | 2583 |  |
| t19 | b1 | 28°43'39" | 83°40'25" | 2529 | mixed coniferous broad leaved forest |
| t18 | b2 | 28°40'50" | 83°36'31" | 2414 |  |
| t17 | T29 | 27°59'20" | 85°58'48" | 2086 | mixed coniferous broad leaved forest |
| t16 | T34 | 27°43'25" | 85°31'36" | 1935 | broad leaved forest |
| t15 | b3 | 28°35'18" | 83°38'54" | 1897 |  |
| t14 | T30 | 27°59'20" | 85°58'48" | 1653 |  |
| t13 | T31 | 27°59'20" | 85°58'48" | 1583 | broad leaved forest |
| t12 | T32 | 27°52'27" | 85°53'37" | 1228 |  |
| t11 | b4 | 28°24'42" | 83°35'58" | 1046 | broad leaved forest |
| t10 | T35 | 27°42'36" | 85°10'57" | 1044 | broad leaved forest |
| t9 | Tp | 27°47'15" | 83°32'27" | 992 | broad leaved forest |
| t8 | T33 | 27°44'51" | 85°50'24" | 804 |  |
| t7 | T42 | 27°47'15" | 83°32'27" | 595 |  |
| t6 | T40 | 27°33'19" | 83°50'32" | 488 | tropical rain forest |
| t5 | T36 | 27°42'43" | 85°10'48" | 217 | tropical rain forest |
| t4 | T37 | 27°46'32" | 84°27'23" | 175 | tropical rain forest |
| t3 | T38 | 27°34'9" | 84°30'50" | 145 | tropical rain forest |
| t2 | T39 | 27°34'9" | 84°30'50" | 144 | tropical rain forest |
| t1 | T41 | 27°33'19" | 83°50'31" | 112 | tropical rain forest |

**Table** **S2** Summary of phytolith types and their source plants and ecoenvironments.

| **Source plant(s) and Ecoenvironment** | **Type** | **Former Names** | **Description** | **Image (Plate)** |
| --- | --- | --- | --- | --- |
| *Palmae* | globular echinate[^1^](#_ENREF_1) | spherical spinose | spherical and beset with prickles[^2-4^](#_ENREF_2) | Fig S1: 1, 2 |
| tropical trees and shrubs | globular granulate[^1^](#_ENREF_1) | Spherical crenate | Spheroid with granular surface[^5^](#_ENREF_5) | Fig S1: 3, 4 |
| evergreen broad-leaved plants | abbreviated stellate[^6^](#_ENREF_6) | Jigsaw-shaped, anticlincal epidermis | different forms of outline with sinuous shape[^6-9^](#_ENREF_6) | Fig S3: 4, 6 |
| broad-leaved plants | cylindrical sclereid[^6^](#_ENREF_6) | sclereid, "Y"-shaped | sclerenchymatous cell, looks like bent elongate | Fig S3: 10, 11 and Fig S4: 10, 11 |
| *Pinaceae* | Gymnosperm |  | There are more than one types, but the most common type is parallelepipedal contorted with a outline of blocky polyhedron[^11^](#_ENREF_11) | Fig S3: 1–3, 5, 8 and Fig S4: 9 |
| *Panicoideae*; warm, humid conditions | bilobate short cell[^1^](#_ENREF_1) | dumbbell | looks like a dumbbell | Fig S1: 7–11, 13 |
|  |  |  |  |  |
| **Table S2** continued |  |  |  |  |
| **Source plant(s) and Ecoenvironment** | **Type** | **Former Names** | **Description** | **Image (Plate)** |
| *Panicoideae*; warm, humid conditions | cylindrical polylobate[^1^](#_ENREF_1) | palylobate | similar to bilobate short cell but owning more than two lobes | Fig S1: 14 |
| Panicoideae | cross |  | has a cross shape; this type produced in maize can be seperated from wild grass by the proportion of morror-image and a larger width (>12.5 μm)[^12^](#_ENREF_12) | Fig S1: 5, 6 |
| *Bambusoideae*; hot, moist climate | oblong concave saddle 1[^6^](#_ENREF_6) | collapsed saddle, long saddle | shaped like a oblong saddle | Fig S1: 15, 16 |
|  | oblong concave saddle 2[^6^](#_ENREF_6) |  |  | Fig S1: 17, 18 |
| mainly in *Chloridoideae* | square saddle[^6^](#_ENREF_6) | short saddle | shaped like a short saddle[^10^](#_ENREF_10) | Fig S1: 19 |
|  |  |  |  |  |
|  |  |  |  |  |
| **Table S2** continued |  |  |  |  |
| **Source plant(s) and Ecoenvironment** | **Type** | **Former Names** | **Description** | **Image (Plate)** |
| *Chloridoideae*, *Panicoideae, Bambusoideae*; warm, humid climate | cuneiform bulliform cell[^1^](#_ENREF_1) | fan-shaped, bulliform | fan-shaped bulliform cell[^8^](#_ENREF_8) | Fig S2: 1–8 |
|  | parallepipedal bulliform cell 1[^1^](#_ENREF_1) | square | bulliform cell with square faces[^8^](#_ENREF_8) | Fig S2: 9–11 |
|  | parallepipedal bulliform cell 2[^1^](#_ENREF_1) | rectangular | bulliform cell with rectangular face[^8^](#_ENREF_8) | Fig S2: 15, 17 |
| *Oryza* | cuneiform bulliform cell-rice | fan-shaped, bulliform | fan-shaped bulliform with crack ornamentation, exclusively from rice[^14^](#_ENREF_14) | Fig S2: 7, 8 |
| *Bambusoideae* | cuneiform bulliform cell-bamboo | fan-shaped, bulliform | fan-shaped bulliform with spine ornamentation[^10^](#_ENREF_10) | Fig S2: 1, 4 |
| spiny cells of grass | hair cell (point)[^1^](#_ENREF_1) | point-shaped | can be subdivided into acicular hair cell  and unciform hair cell[^1^](#_ENREF_1) | Fig S2: 13, 14 and 16 |
|  |  |  |  |  |
| **Table S2** continued |  |  |  |  |
| **Source plant(s) and Ecoenvironment** | **Type** | **Former Names** | **Description** | **Image (Plate)** |
| Pooideae; cold, arid climate | rondel[^1^](#_ENREF_1) | hat-shaped | conical | Fig S1: 20, 21, 25 |
|  | trapeziform[^1^](#_ENREF_1) | trapezoid | trapeziform cell produced in grass | Fig S1: 30–33 |
|  |  |  |  |  |
| *Stipa*, cold, arid areas | *stipa*-bilobate short cell |  | Has slim neck and differing opposite sides, the top and bottom are dumbbell but the latter is bigger, so lateral side is trapezoid[^15^](#_ENREF_15) | Fig S1: 12 |
|  | gobbet | nubby-irregular |  |  |
| *Cyperaceae*; wet places | sedge | papillae | polyhedron outline, central projection[^8^](#_ENREF_8) | Fig S1: 34–37 |
| ferns | pteridophyte |  | elongate with triangular cross section[^10^](#_ENREF_10) | Fig S2: 12 and Fig S4: 1, 3 |
| cover wide distribution of grasses | elongate smooth |  | smooth surface[^10^](#_ENREF_10) | Fig S4: 6, 7 |
|  | elongate echinate |  | echinate surface[^10^](#_ENREF_10) | Fig S4: 2, 4 |
|  |  |  |  |  |
| **Table S2** continued |  |  |  |  |
| **Source plant(s) and Ecoenvironment** | **Type** | **Former Names** | **Description** | **Image (Plate)** |
| ecological characteristics still unknown | one-horned tower[^17^](#_ENREF_17) |  |  | Fig S1: 26, 27 |
|  | two-horned tower[^17^](#_ENREF_17) |  |  | Fig S1: 28, 29 |
| not yet investigated | unknown |  |  | Fig S5: and Fig S3: 9 |

**Table S3** Classification function coefficients of canonical discriminant functions.

| **Phytolith Type** | **Group** | | | | |
| --- | --- | --- | --- | --- | --- |
|  | 1 | 2 | 3 | 4 | 5 |
| parallepipedal bulliform cell 1 | 10.783 | 12.151 | 12.514 | 11.823 | 9.968 |
| parallepipedal bulliform cell 2 | 5.975 | 6.322 | 6.324 | 7.833 | 8.164 |
| elongate smooth | 9.259 | 9.801 | 10.239 | 12.775 | 12.000 |
| elongate echinate | 4.581 | 3.994 | 4.691 | 4.910 | 5.567 |
| bilobate short cell | 9.604 | 10.815 | 10.324 | 9.941 | 8.757 |
| *stipa*-bilobate short cell | 9.826 | 10.590 | 10.909 | 13.318 | 12.062 |
| oblong concave saddle 2 | 10.770 | 12.239 | 12.240 | 13.550 | 12.313 |
| square saddle | .765 | -.615 | 1.013 | -3.080 | -2.917 |
| oblong concave saddle 1 | 4.453 | 3.916 | 3.848 | 4.309 | 4.388 |
| cuneiform bulliform cell | 4.426 | 4.594 | 4.337 | 4.232 | 3.868 |
| rondel | 10.044 | 10.239 | 11.135 | 15.204 | 15.594 |
| cylindrical sclereid | 14.303 | 14.568 | 14.865 | 15.104 | 13.608 |
| hair cell | 8.789 | 8.731 | 9.235 | 13.399 | 15.025 |
| trapeziform | 9.548 | 9.915 | 10.392 | 13.236 | 13.435 |
| globular | 8.070 | 6.285 | 6.220 | 6.313 | 5.726 |
| Gymnosperm type | 9.715 | 10.330 | 10.539 | 12.123 | 10.520 |
|  |  |  |  |  |  |
| **Table S3** continued |  |  |  |  |  |
| gobbet | 14.347 | 14.684 | 15.471 | 19.450 | 20.170 |
| (Constant) | -359.235 | -400.223 | -416.605 | -554.911 | -546.181 |
| Fisher's linear discriminant functions | | | | | |


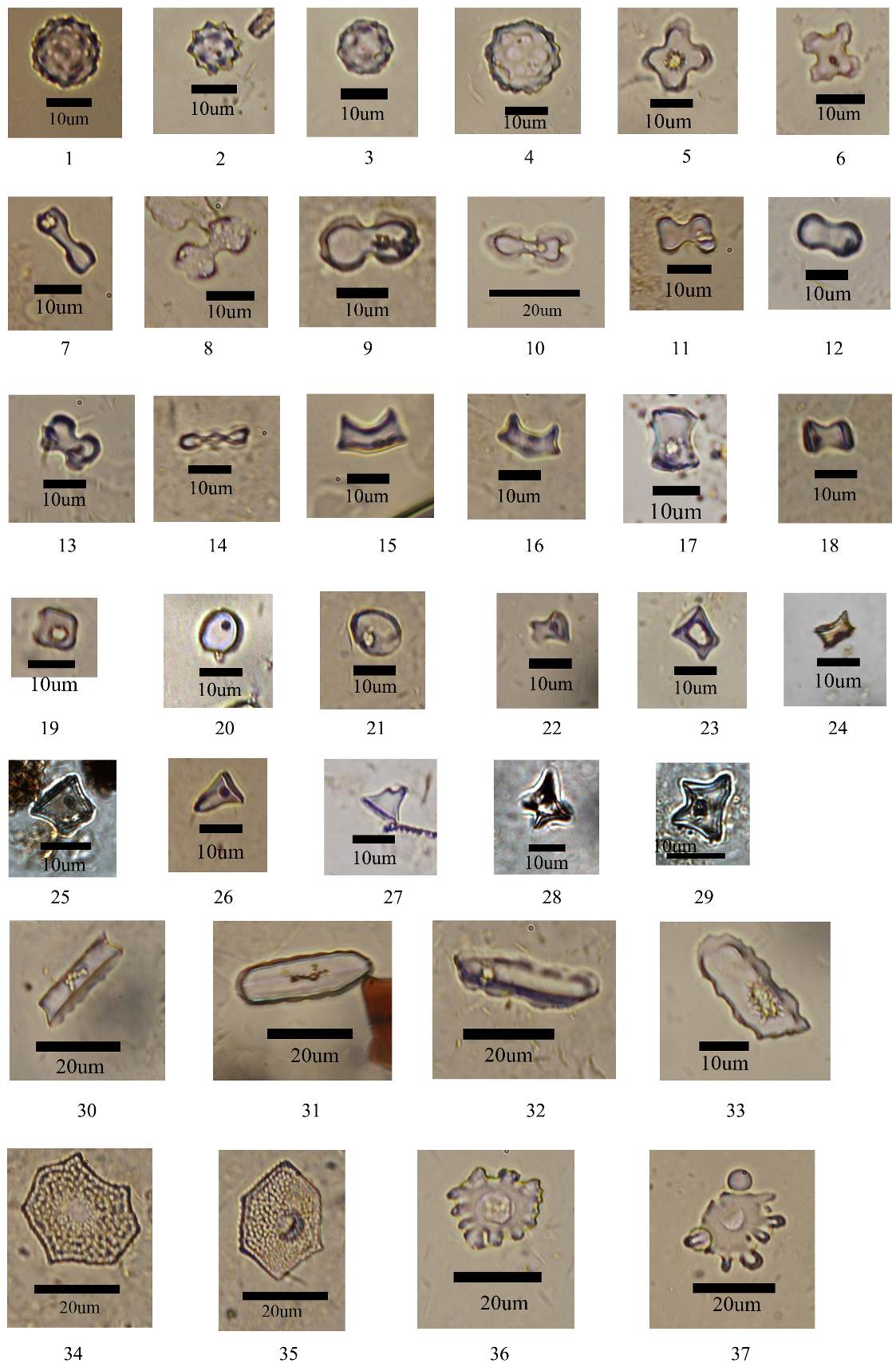
**Figure S1** Phytolith types in surface soils from the southern Himalaya (1).

1–4 globular; 5 cross (maize); 6 cross (wild grass); 7–11, 13 bilobate short cell; 12 *stipa-*bilobate short cell; 14 palylobate short cell; 15, 16 oblong concave saddle 1; 17,18 oblong concave saddle 2; 19 Square saddle; 20–25 rondel; 26, 27 one-horned tower; 28, 29 two-horned tower; 30–33 trapeziform; 34–37 sedge.


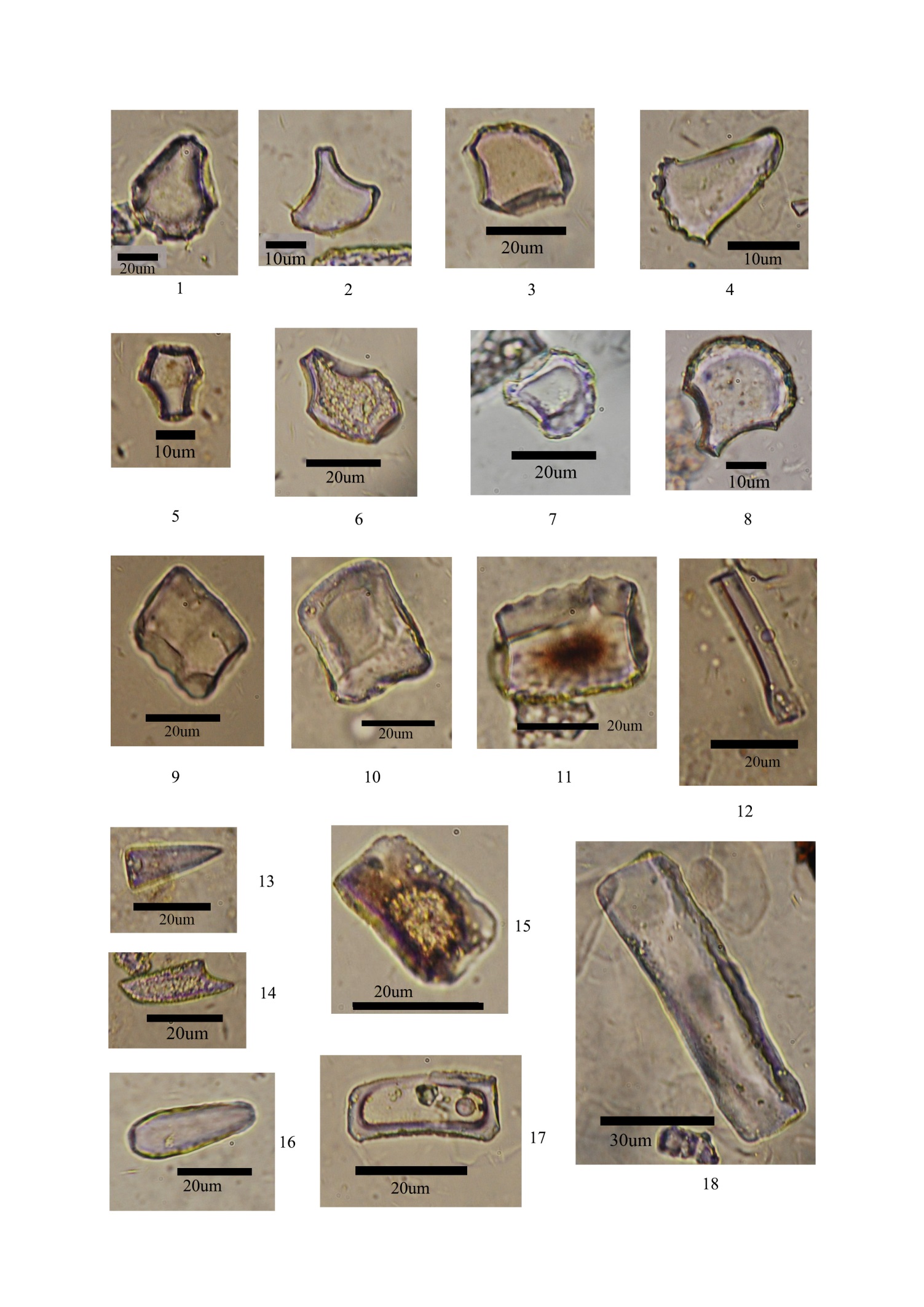
**Figure S2** Phytolith types in surface soils from the southern Himalaya (2).

1–8 cuneiform bulliform cell; 1, 4 cuneiform bulliform cell-bamboo; 7, 8 cuneiform bulliform cell-rice; 9–11 parallepipedal bulliform cell 1; 12 pteridophyte type; 13, 14, 16 hair cell; 15, 17 parallepipedal bulliform cell 2; 18 elongate.


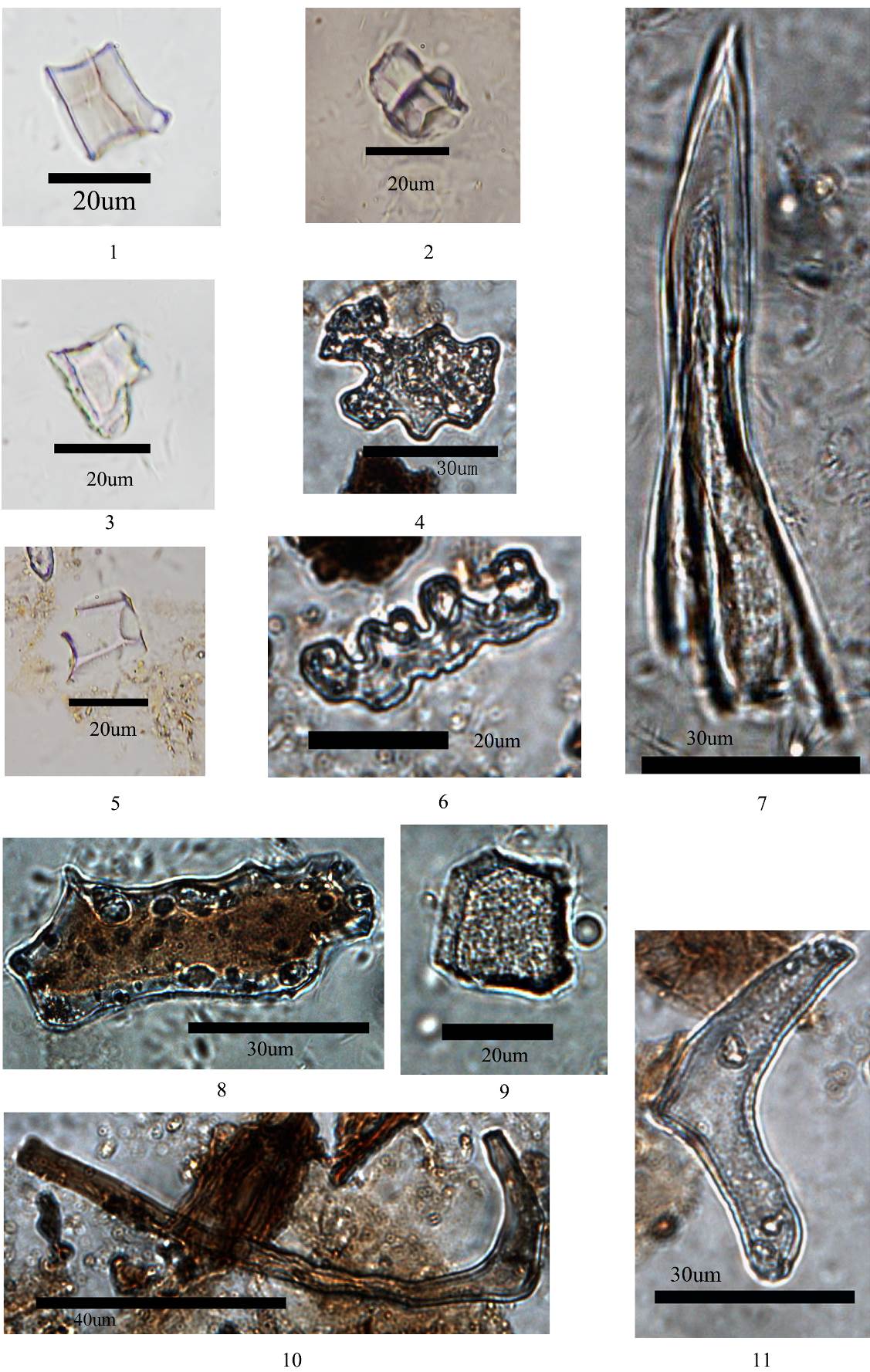
**Figure S3** Phytolith types in surface soils from the southern Himalaya (3).

1, 2, 3, 5 gymnosperm types; 4, 6 abbreviated stellate; 7 beak; 9 dicotyledonous blocky; 10, 11 cylindrical sclereid.

**
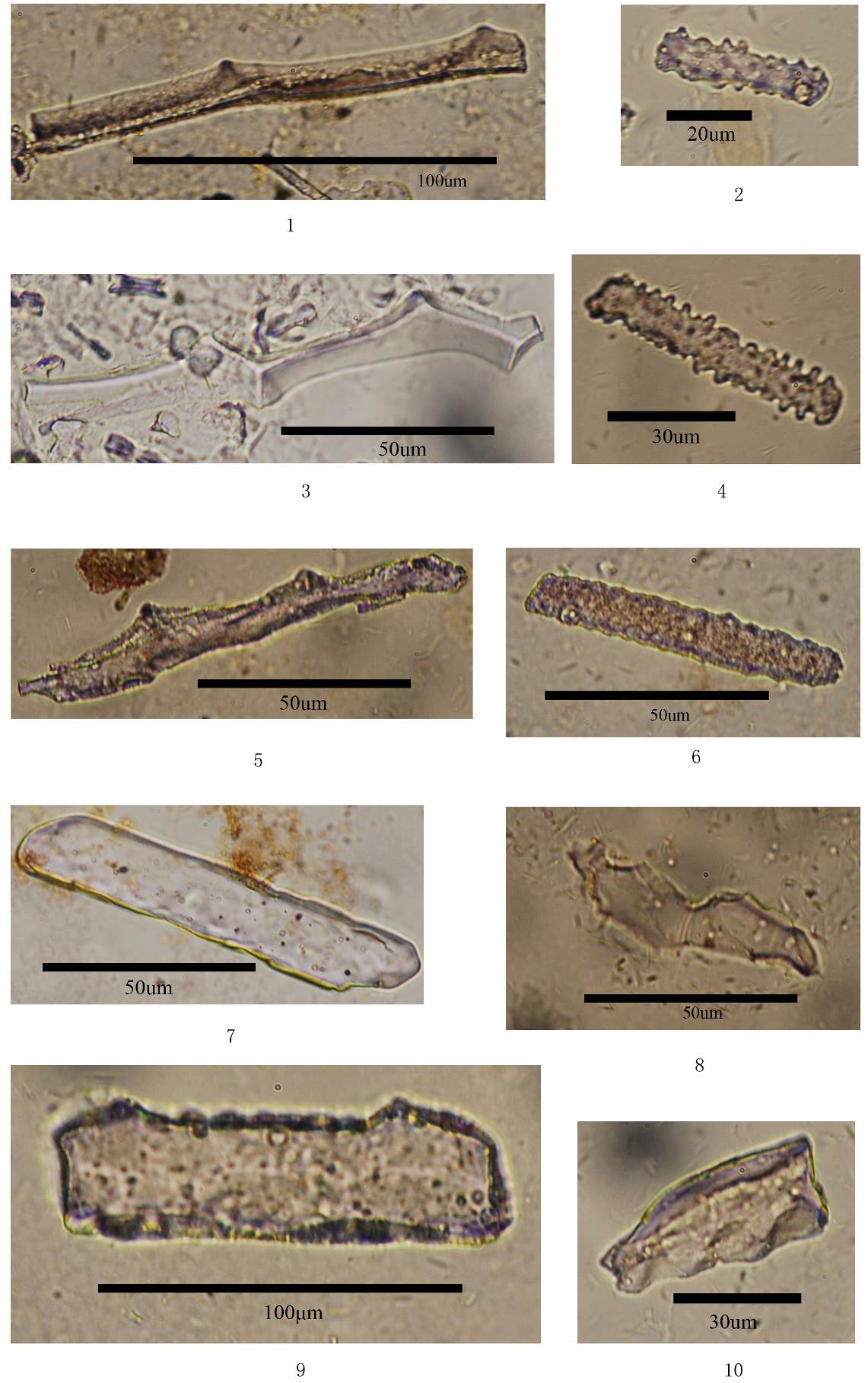
**

**Figure S4** Phytolith types in surface soils from the southern Himalaya (4).

1, 3 pteridophyte types; 2, 4 elongate echinate; 5, 8, 10 broadleaved type; 6, 7 elongate smooth; 9 gymnosperm types.


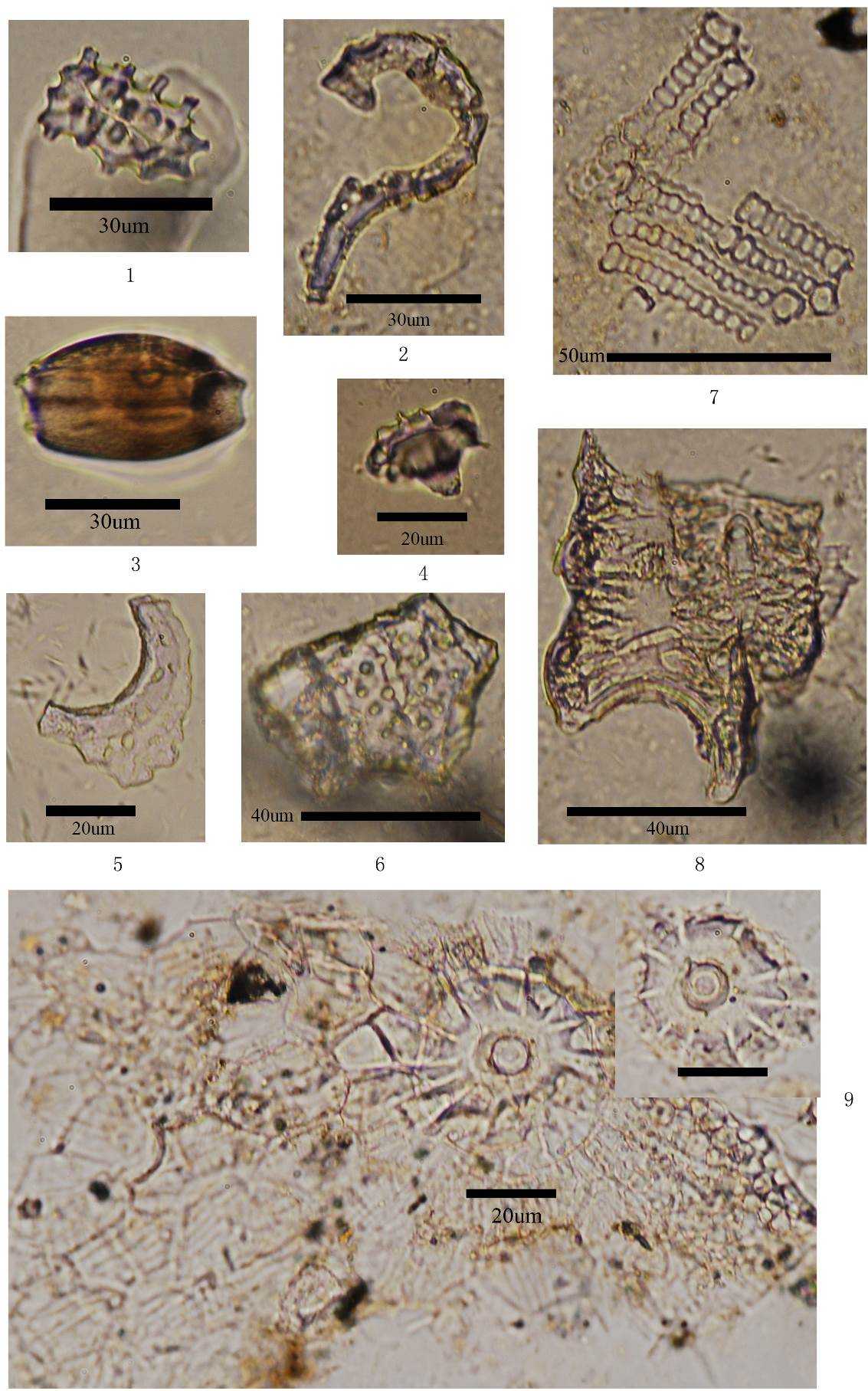
**Figure** **S5** Unknown phytolith types in surface soils from the southern Himalaya.

# References:
